# Plant Calcium Content: Ready to Remodel

**DOI:** 10.3390/nu4081120

**Published:** 2012-08-21

**Authors:** Jian Yang, Tracy Punshon, Mary Lou Guerinot, Kendal D. Hirschi

**Affiliations:** 1 United States Department of Agriculture/Agriculture Research Service Children’s Nutrition Research Center, Department of Pediatrics, Baylor College of Medicine, Houston, TX 77030, USA; Email: jiany@bcm.edu; 2 Department of Biological Sciences, Dartmouth College, Hanover, NH 03755, USA; Email: tracy.punshon@dartmouth.edu (T.P.); mary.lou.guerinot@dartmouth.edu (M.L.G.); 3 Vegetable and Fruit Improvement Center, Texas A&M University, College Station, TX 77845, USA

**Keywords:** calcium, bioavailability, biofortification, bone mineralization, synchrotron X-ray fluorescence (SXRF), oxalate, antinutrient

## Abstract

By identifying the relationship between calcium location in the plant cell and nutrient bioavailability, the plant characteristics leading to maximal calcium absorption by humans can be identified. Knowledge of plant cellular and molecular targets controlling calcium location in plants is emerging. These insights should allow for better strategies for increasing the nutritional content of foods. In particular, the use of preparation-free elemental imaging technologies such as synchrotron X-ray fluorescence (SXRF) microscopy in plant biology may allow researchers to understand the relationship between subcellular location and nutrient bioavailability. These approaches may lead to better strategies for altering the location of calcium within the plant to maximize its absorption from fruits and vegetables. These modified foods could be part of a diet for children and adults identified as at-risk for low calcium intake or absorption with the ultimate goal of decreasing the incidence and severity of inadequate bone mineralization.

## 1. Introduction

The three most important factors in buying a home are, location, location, location. We would like to co-opt this adage to help communicate the concepts surrounding nutrient partitioning in plants. Throughout this review we will make comparisons between nutrient partitioning in plants and real estate. The concepts of investing, renovating, and knowing your neighbors are all important for both real estate agents and plant biologists trying to improve the nutritional quality of foods.

**Figure 1:**
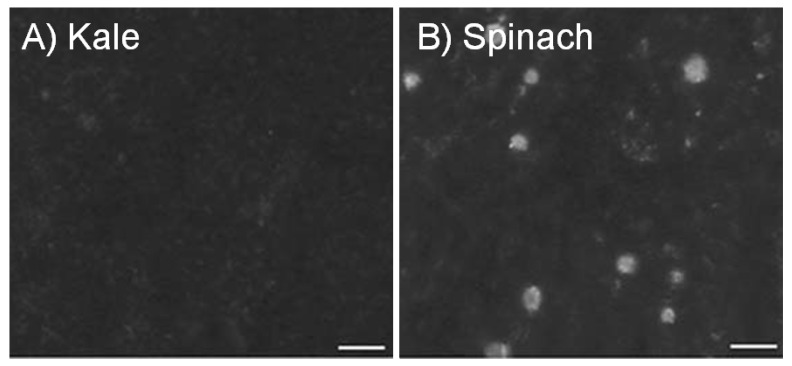
Calcium oxalate in kale and spinach. Leaves from kale (**A**) and spinach (**B**) were purchased at a local grocery store, cleared of their chlorophyll, and visually inspected using a light microscopy [7]. A representative portion of each leaf is shown using partially polarized light. Ca-oxalate crystals appear as bright spots. Note, spinach sequesters ample amounts of Ca as the oxalate salt. This is in sharp contrast to kale that does not appear to form these crystals. Images were captured using a CCD72 camera. Bar = 100 µm. Note: This figure is adapted with permission from [7]. Copyright © 2007, Springer Science+Business Media B.V.

Making a good relationship with our neighbors is important. The better the neighborhood the more valuable the homes become. Much like we live in a community, plant nutrients are often found associated with neighboring complexes [1]. Calcium (Ca) in plants is primarily complexed with oxalate, phytate, fiber, lactate, fatty acids, protein, and other compounds [2,3]. Although many edible plants are high in total Ca, complexation with oxalate (forming Ca-oxalate crystals) renders it undigestible (Figure 1) [4,5,6], resulting in oxalate being considered an “antinutrient”. Soluble oxalates can also reduce absorption of minerals from other consumed foods [3].

Calcium absorption is inversely proportional to oxalic acid content in food [4,8,9,10]. Although spinach contains 23.8 to 26.7 mg/g Ca, the oxalate content is high (105.2 mg/g) and as a result the Ca bioavailability is low; kale, however contains 26.3 to 27.6 mg/g Ca but has low (2.8 mg/g) oxalate levels and so bioavailable Ca is high (Figure 1) [4]. A notable exception to this is soybeans, where both oxalate levels (35 mg/g [11]) and bioavailable Ca are high [11]. The aim of the review is to summarize the current state of our knowledge of parameters affecting Ca bioavailability in crop plants. We discuss approaches and issues surrounding the alteration of Ca content to improve bioavailability. We then showcase how an elemental analysis tool synchrotron X-ray fluorescence (SXRF) microscopy can be used to improve our understanding of nutrient availability. We conclude with some thoughts concerning the future use of engineered foods to improve Ca uptake. 

## 2. Calcium in Foods, Fortification and Supplements

Keeping with our real estate theme, there are always ways to improve the landscape and to increase value for your property. Likewise, multiple methods are available to achieve adequate dietary Ca intake, such as changing the surroundings of the Ca^2+^ ion to increase its bioavailability. 

Since the bioavailability of Ca varies greatly among foods, nutritional information listing the total amount of Ca is misleading [12]. A simple system is presented here to assess foods that are reasonable Ca sources (Table 1). For a food to be a good Ca source, two criteria must be met: a standard serving must contain at least 30 mg absorbable Ca and less than 418 kJ (100 kcal) of the food must provide 30 mg absorbable Ca [13].

**Table 1:** Food sources of Calcium [14].

Food	Serving	Ca per Serving (mg)	Ca per 418 kJ (100 kcal) (mg)	Ca AbsorbServing (mg)	Energy per Serving (kJ)	Ca Absorp(%) ^B^
Orange Juice 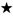 ^,A^ (calcium fortified CCM)	237 mL	300	268	90	468	30
Milk 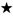 (whole)	237 mL	276	189	89	610	32
Kale ^○^	85 g	47	448	23	46	49
Spinach ^○,B^	85 g	122	595	6	88	5
Soybean ^●^ (cooked)	86 g	88	59	21	623	24
Carrots ^○^ (raw, sliced)	1 cup	42	52	22	221	53
Potato ^●^ (baked)	1 med	26	161	6	675	22 ^□^

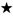
 Foods that meet both criteria for a good calcium source (Provides at least 30 mg of absorbable calcium per serving and per 418 kJ (100 kcal) of the food); ^○^ Foods that meet only one of the two criteria for a good calcium source (Provides at least 30 mg of absorbable calcium per serving or per 418 kJ (100 kcal) of the food); ^●^ Foods that do not meet either of the two criteria for a good calcium source (Provides neither 30 mg of absorbable calcium per serving or per 418 kJ (100 kcal) of the food); ^□^ Estimated fractional absorptions from [15]; **^A^** Calcium content can vary greatly depending on the brand; CCM, calcium citrate-malate; **^B^** Cooked by boiling or steaming.

Dairy products are the most significant source of Ca (Table 1) [15]. Green leafy vegetables such as broccoli and bok choy are also good Ca sources. Calcium is also added to food products such as bread and orange juice [16]. Dietary Ca supplements can be an effective means of delivering Ca but each of these sources has its limitations, and developing high Ca foods is still the best route to obtain adequate Ca nutrition. For example, many consumers do not drink enough milk or eat enough dairy products to provide sufficient Ca. Lactose intolerance, or at least the perception of intolerance, affects between 30 and 50 million Americans and limits dairy consumption [17,18]. Fortified foods are problematic because among certain groups, a lack of awareness of Ca malnutrition and low consumer demand thwarts the use of this technology worldwide [19,20]. Furthermore, in developing countries, supplement distribution systems are inadequate and regulatory systems are often not in place to successfully implement fortification. Like fortification efforts, Ca supplements may not always work because compliance is low [21,22,23,24]. For example, CaCO_3_ tablets must be taken with food so stomach acid can facilitate absorption, but Ca-citrate tablets do not have this limitation. Unfortunately, Ca supplements will only work for informed and affluent consumers, and are far less likely to be effective in disadvantaged areas. 

## 3. Biofortification: Altering Calcium Content of Plant-Based Foods

The aim of Ca biofortification strategies is to increase Ca content of plants without adversely affecting plant growth or increasing production costs [25,26]. Strategies to increase Ca content of plants could conceivably include: (1) increasing Ca supply to cells; (2) increasing Ca uptake into cells; (3) removing compounds that render Ca unavailable and/or (4) increasing Ca storage at the cellular and/or tissue level.

The ability to manipulate beneficial qualities of plant-based foods has been transformed by biotechnology. Molecular biology techniques can be concentrate elements into the edible portion of crop plants, in effect remolding crops to house increased nutrients [27]. In 2008 Copenhagen Consensus, a panel of the world’s foremost economists deemed plant biofortification, the process of increasing the bioavailable concentration of a nutrient element in foods, as one of the preeminent global challenges. Furthermore, economists predicted tremendous benefits compared to costs associated with developing the technology [27,28]. Due to low genetic variability for micronutrient levels in food crops, nutritional enhancement of crops through conventional breeding is often limited, and molecular approaches can be a valid alternative. Nutritional genomics studies the relationship between genomes, nutrition and health [27,29,30]. The ability to rapidly identify and characterize gene function and engineer genes to alter plant metabolism is a driving force in biofortification efforts [31].

One way to increase Ca uptake by plants would be to increase mass flow of water (and consequently Ca) to the roots. This could be achieved by increasing water demand from the plant; however this is not an ideal strategy because fresh water is generally limiting, especially in developing countries. Alternative options for increasing Ca supply to plant tissues would be to increase the demand for Ca, by increasing cell wall Ca binding or entry of Ca into cells [32].

The cation-binding capacity (CEC) (the maximum quantity of total cations, of any class, that the cell wall is capable of holding, which are available for exchange with surrounding solutes) of the cell wall represents another potential target for increasing the Ca content of plant tissues [33]. The CEC of different tissues and plant species varies widely [34]; indicating a genetic basis for CEC regulation. Furthermore, the CEC of cereals is generally low and this has been correlated with the low Ca content in shoots [33]. Increasing the CEC of roots may be a strategy to get more Ca into plants. However, processes that regulate Ca signaling are poorly understood as are the mechanisms by which Ca can affect cell wall function [35]. Therefore, any such strategy to modify cell wall Ca binding capacity may produce undesirable side-effects. The transport of Ca into the cell is mediated by various ion channels and transporters and it is tightly regulated. The overexpression of these channels or transporters is likely to disturb normal cell functions due to the importance of maintaining cytosolic Ca levels. However, by overexpressing transporters at internal Ca stores such as the vacuole, we may indirectly increase Ca entry into cells. 

These examples highlight the potential strategies that could be used to alter Ca content in crops. We will focus predominately on the removal of antinutrients and the increased expression of endomembrane Ca transporters as a means to biofortify crops with increased Ca.

### 3.1. Removing Antinutrients: Less Is More

A teardown is a process in which one buys a home, demolishes and replaces it with an improved model. Antinutrients are a promising target for engineering molecular “teardowns”. Calcium complexed with oxalate is unavailable for absorption across the gut [4,7], therefore applying this renovation concept to plant nutrient bioavailability involves uncoupling Ca and oxalate. Plant species can be divided into species that contain soluble oxalate and species that deposit Ca-oxalate crystals in their vacuoles, which impacts their abilities to accumulate Ca [33]. Studies have used *Medicago truncatula* (a forage legume similar to alfalfa) to manipulate Ca-oxalate crystal formation. Humans do not consume Medicago; however, the plant contains Ca-oxalate crystals in the leaf tissue like those found in other plant foods such as spinach and it is an ideal model plant system for legumes with its short generation time, small genome, and ease of genetic manipulation [36]. 

**Figure 2:**
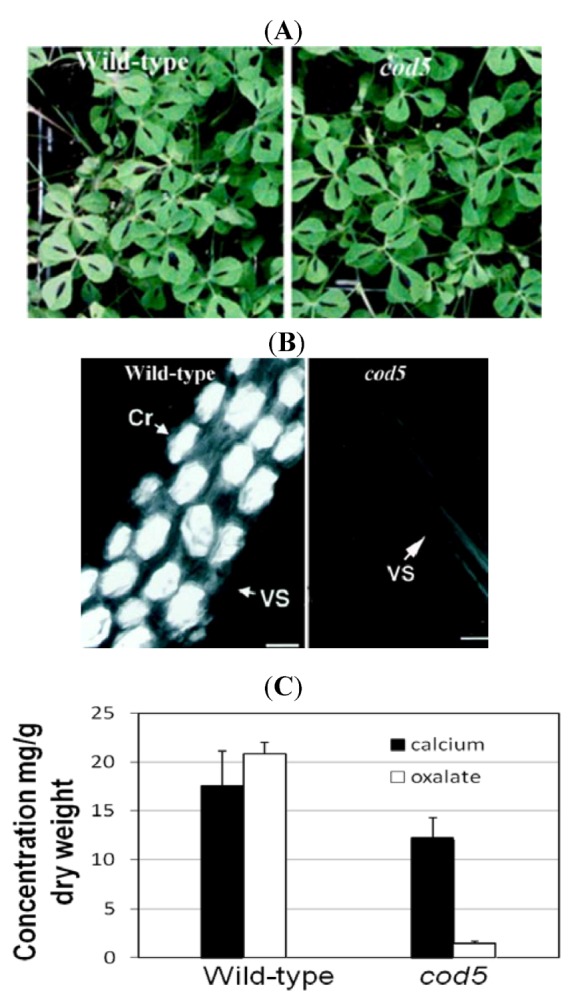
Characterization of *calcium oxalate deficient* (*cod5*) plants. (**A**) Wild-type and *cod5* plants grown in soil display similar plant phenotypes. (**B**) Whole-mount leaf clearing showing cells from wild-type and *cod5* plants. The crystals appear as bright prismatic structures using polarized light. Scanning electron micrographs confirm the absence of crystals in the mutant Cr: Ca-oxalate crystal; VS: Vascular strand. (**C**) Ca and oxalate levels were measured in leaves harvested from wild-type and *cod5* plants. Although Ca levels were similar between the two plants, the amount of Ca they each sequestered as the oxalate salt varies drastically [38]. Note: Figure 2A,B are adapted with permission from [37]. Copyright © 2000, American Society of Plant Physiologists.

The *Ca oxalate deficient 5* (*cod5*) mutant was isolated from a chemical mutagenesis screen, and is devoid of Ca-oxalate crystals [37] (Figure 2). Oxalate may still be present as soluble sodium and potassium salts. Thus, although *cod5* does not have Ca-oxalate crystals, other forms of oxalate salts are still found in the *cod5* mutants. Growth studies and composition measurements indicate no detectable difference between *cod5* and wild type with the exception of the presence of oxalate crystals. These findings suggest that it may be possible to alter the function of a single gene in a crop such as spinach to remove Ca-oxalate crystals, and leave all other processes unaffected. Based on studies in Medicago (Figure 2), Ca-oxalate deficient spinach plants may grow as vigorously as the unmodified varieties.

Another major antinutrient of Ca in plants is phytic acid (known as inositol hexakisphosphate (IP6), or phytate when in salt form). It is the storage form of phosphorus in many plant tissues, especially bran of cereals [39,40]. Humans and nonruminant animals cannot digest phytate. Moreover, it chelates and thus makes unabsorbable certain important minerals such as zinc (Zn) and iron (Fe), and to a lesser extent Ca and magnesium (Mg). Phytate concentrations are highest in unrefined cereals and legumes (approximately 600 mg/100 g dry weight), followed by refined cereals (approximately 100 mg/100 g dry weight) and then starchy roots and tubers (<20 mg/100 g dry weight) [41]. If foods with high phytate content dominate the diet, fortification is required for optimal health. Dephytinization, either in the household or commercially (mainly by removing the phytate-rich tissue layers in unrefined cereals by steeping in water), can potentially enhance mineral absorption in high-phytate foods, although probably not enough to overcome the shortfalls in Fe, Zn, and Ca content of plant-based foods used in developing countries [42]. The World Health Organization suggests that dephytinization must be combined with enrichment with animal-source foods and/or fortification with appropriate levels and forms of mineral fortificants in order to ensure proper health [43]. 

Genetic engineering by creating low-phytic acid (lpa) crops has had some success in increasing bioavailable Fe, Zn and Ca levels [44]. However, phytic acid has some health promoting properties and important roles in plant growth and development, so simply removing it can have unintended “collateral” damage to health and crop productivity [45,46]. Future genetic engineering approaches may focus on creating specific lpa lines that are phytic acid deficient only in the edible portions of the foods. 

### 3.2. Manipulating Transporters: Mining Ca from Soil

**Figure 3:**
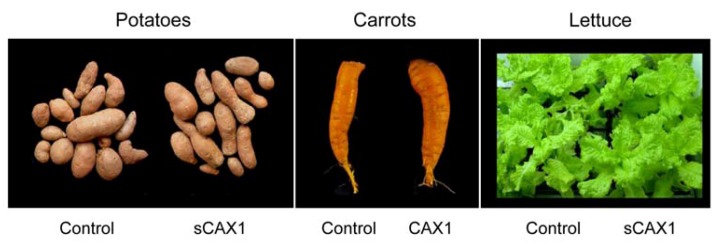
CAX1 expression in a variety of popular vegetables. Control and CAX1-expressing plants are indistinguishable in terms of yield, oxalate levels and growth characteristics; however, the amount of calcium is increased in the transgenic lines [27].

Calcium must enter the plant before it can be partitioned within a plant cell. Increased bioavailable mineral content in agriculturally important crops can be engineered through manipulating plant mineral transporters [47,48]. Ca/H^+^ antiporters, located on the vacuolar membrane, are known to be important for Ca sequestration [49]. We hypothesized that an engineered version of a plant Ca/H^+^ antiporter (e.g., CAX1) could be used for biofortification, by increasing bioavailable Ca levels within edible roots, such as carrots. Although there has been some work with other Ca transporters, altering their expression has not proven to be as effective as the *CAX* genes [50]. We have modified carrots to express high levels of a deregulated *Arabidopsis* Ca/H^+^ antiporter (sCAX1), resulting in accumulation of almost two-fold more Ca in the edible part compared to control plants, without affecting growth, development or fertility, under controlled lab conditions [27]. Using this approach, rice, lettuce, carrots, and potatoes lines have all been engineered with increased Ca content (Figure 3). In the potatoes, carrots and lettuce we have verified that the increased Ca content is caused by expression of a single copy of the *CAX*1 gene. In carrots, we have used genetic crosses to verify that this trait is heritable [51]. Using both biochemical analysis and microscopic studies, we found no alteration in oxalate levels, the antinutrient discussed earlier.

### 3.3. Feeding Studies Are Essential To Validate Biofortification Efficacy

Biofortified foods offer nutritional solutions for the most vulnerable human populations: women, infants and children in developing countries, on primarily plant-based subsistence diets. One of the first steps in the development of these foods is to demonstrate their purported bioequivalence and bioavailability in their nutritional value compared to traditional foods. However, few studies have rigorously tested the nutritional quality of biofortified foods and measured the parameters that determine the eventual successes of conventionally bred foods or genetically modified lines [52]. In the case of ‘golden’ rice which was engineered to contain more β-carotene [53], there was an initial delay in verifying its nutritional benefits through feeding studies [54]. The recent human feeding studies will prove critical in its eventual marketing success. A notable exception to the lack of nutritional studies is the work manipulating the antinutrient phytic acid in crops [42,55,56]. These studies serve as a model for the integration of plant biology and human nutritional studies.

At the Children’s Nutrition Research Center (CNRC) at Baylor College of Medicine we have performed feeding studies with modified foods. In an animal feeding study, we demonstrated that diets that contain *cod5* have 22.8% higher Ca absorption than diets containing wild-type Medicago [7]. This suggests the removal of antinutrients is an effective way to partially enhance bioavailability of Ca. As mentioned earlier, these *cod5* lines still have soluble oxalate and this may account for the remaining Ca that is not bioavailable to the animals. These feeding studies have also been performed on herbivorous insects, where the changes are dramatic. Mineral crystals of Ca-oxalate in leaves of Medicago are effective deterrents of insect feeding, and they inhibit conversion of leaves into insect body mass after consumption [57]. Growth of the insects is greater on *cod* mutants of *Medicago truncatula* with decreased crystal accumulation. Insects feeding on *Medicago truncatula* with Ca-oxalate crystals experience negative effects on growth and mandible wear compared to those feeding on artificial diets amended with smaller crystals. This is a valuable insight, as increased bioavailability to insects means reduced yield. This negative consequence must be carefully considered before applying these approaches to production agriculture.

With the Ca transporter (sCAX1) enhanced foods, feeding trials using these labeled carrots demonstrated that the total amount of Ca absorbed was significantly increased, in both mice and humans, with diets containing the modified carrots [47]. In the human feeding studies, Ca absorption efficiency was 42.6% ± 2.8% and 52.1% ± 3.2% (*p* < 0.001) for the sCAX1-carrots and control carrots, respectively; however, total Ca absorption per 100 g of carrots was 41% ± 2% higher for sCAX1-carrots compared to control carrots (26.50 *vs.* 15.34 mg Ca/100 g, *p* < 0.001) [47]. Interestingly, not all the increased Ca in the transporter-modified carrots was bioavailable. This may be due to a fraction of the extra Ca being bound to antinutrients within the carrot. This serves as a cautionary example against assuming that all increases in nutrient content directly equate to increased bioavailability. However, the modified carrots did prove to be a better source of Ca because total Ca absorbed was higher. We postulate that the Ca content has increased within the vacuoles of the modified carrots, and are in the process of directly imaging the Ca redistribution in these plants to test this hypothesis. 

*CAX*-expressing lettuce has been analyzed for taste and flavor components compared to non-transgenic controls [58]. Professional descriptive panelists were used to determine attributes relevant to flavor, bitterness and crispness of control and the s*CAX*1-expressing biofortified lettuce. The flavor of the biofortified lettuce was virtually identical to the controls and no effect was noted on bitterness and crispness. These studies suggest that consumer acceptance of the biofortified lettuce should not be affected based on a change in flavor, bitterness or crispness since statistically no significant differences were observed.

## 4. Analysis of the Mineral Distribution, Abundance and Speciation within Food Crop Plants

Online real estate started using spatial technology by putting points on a map. Today, real estate web sites offer advanced spatial features and related geographical tools to aid home buyers. Similarly, techniques now exist to allow plant biologists to map the distribution and abundance of elements within plant tissues [59,60]. In contrast to bulk measurements, these techniques show the confinement of elements within specific plant organs, tissues, cells, cell membranes and even organelles. This provides information about the role and bioavailability of the element(s), and the function of the responsible transport systems. These techniques include inductively coupled plasma-mass spectroscopy (ICP-MS) [61], X-ray micro-analysis (XMA) coupled with cryo-scanning electron microscopy [62], and SXRF microscopy. ICP-MS allows for measurements of the ionome in specific tissue types but normally does not provide cellular or subcellular resolution. XMA offers subcellular resolution but requires sectioning of tissues. SXRF microscopy does not require sectioning and can provide high resolution. In this section, we will highlight the use of SXRF microscopy in elemental analysis.

### 4.1. Elemental Imaging Using Synchrotron X-ray Fluorescence (SXRF) Microscopy

The potential of synchrotron X-rays in elemental imaging of plant tissues has been realized in recent years [63,64,65]. This technique uses the high-energy, focused X-rays generated at synchrotron facilities to image primarily the first row transition elements (e.g., Manganese, Iron, Cobalt, Nickel, Chromium, Copper, Zinc) in a wide variety of tissues, ranging from dry seed to hydrated roots. In comparison with bench-top X-ray fluorescence instruments, synchrotron-based techniques offer lower detection limits and more focused beams. Increasing use of SXRF in plant science has followed advances in detector sensitivity, stage motor design and faster data acquisition, all of which have made hydrated (and in some cases living) plant analysis possible [66]. 

In particular, SXRF has been successfully used as part of the plant molecular genetics toolbox. Ion-responsive stains and fluorophores are extensively used in molecular genetics to show alterations in metal distribution as a result of gene disruption. However, SXRF offers non-destructive, simultaneous quantitative imaging of a wider range of elements (than those for which ion-sensitive probes exist), without the need for intrusive sample preparation. Furthermore, SXRF imaging reveals all forms of the element of interest, regardless of their solubility or oxidation state. Plant lines altered in various types of membrane-intrinsic transport proteins are particularly amenable to analysis by SXRF imaging [65], because these transporters are responsible for selective metal partitioning between cytoplasm, membranes, vacuoles and other subcellular organelles.

### 4.2. Application of SXRF Imaging to the Study of Ca Transporters

The multiple roles that Ca plays in the cell [67] suggest that the localization, abundance and speciation differ among the different Ca compounds involved in each of these roles. It is, as a result, particularly more challenging to visually distinguish changes in Ca, because of its numerous roles and macronutrient level abundance. For this reason, Ca is most accurately measured in fresh, fully hydrated or preferably living tissue, and SXRF is one of the few spatially resolved analytical techniques able to do this. 

#### 4.2.1. SXRF Microscopy Using Dehydrated Tissues

We have used SXRF to investigate changes in Ca distribution and abundance attributable to the vacuolar membrane antiporters, CAX1 and CAX3 [63] introduced earlier. Expression profiles suggested that likely phenotypes would be detected in cotyledons and seed, the latter as a result of higher expression during silique development. We focused our initial analysis on dry mature seed, due to the greater stability of seed tissues for the extended time periods that were previously required for SXRF analysis. This showed that maximum pixel Ca abundances were higher in the seed coat of single loss-of-function mutants *cax1-1* and *cax3-1* but lower in the *cax1/cax3* double mutant and a line overexpressing a truncated version of *CAX*1 (“short”, s*CAX*1), where the regulatory region had been removed, under the control of the 35S promoter, known as 35S-s*CAX*1 [63]. Since these antiporters remove Ca from the cytoplasm (in exchange for H^+^), the presence of more Ca in the seed coat of the single loss-of-function mutants might suggest either that their role is to transport Ca out of maternal tissues that later become the seed coat during seed development, and/or that up-regulation of other cation transporters over-compensate for the loss of Ca transport. 

#### 4.2.2. SXRF Microscopy Using Hydrated Tissues

**Figure 4:**
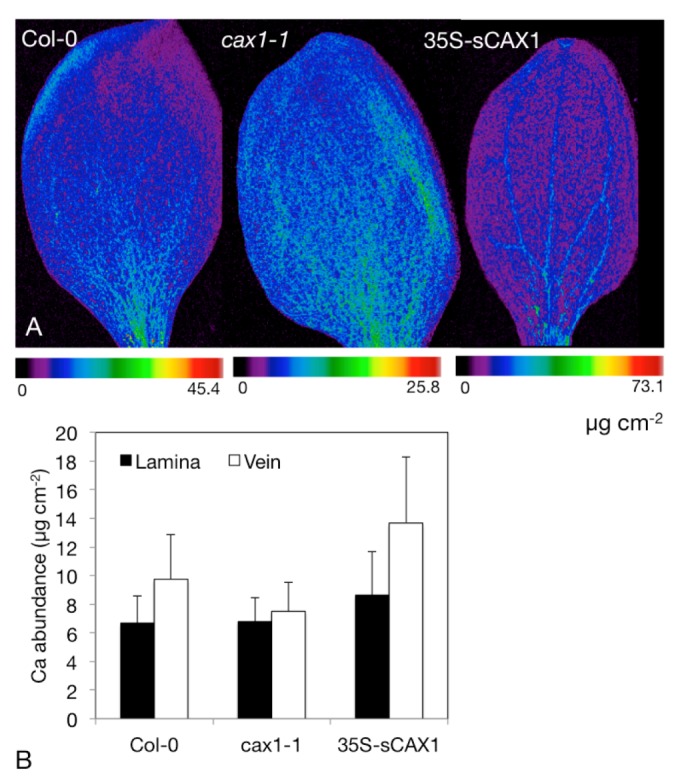
Synchrotron X-ray Fluorescence (SXRF) analysis of Ca content in *Arabidopsis* cotyledons. (**A**) SXRF images of Ca in 9-day old *Arabidopsis* cotyledons from plant grown on full strength B5 medium. Images are individually scaled. (**B**) Region of interest analysis on Ca abundance in the veins and lamina, showing mean and standard deviation of the image shown in panel A.

Expression of CAX1 is strongest in the cotyledons which we have also imaged via SXRF. Figure 4 shows Ca distribution and abundance (expressed as µg cm^−2^) in cotyledons of *Arabidopsis thaliana* from the wild-type background (Columbia-0), *cax1-1* and the overexpressor 35S-s*CAX*1. These plants were germinated and grown on full-strength B5 solid medium for 9 days, and imaged at Stanford Synchrotron Radiation Lightsource beamline BL2-3 at 11 keV, using a 2 × 2 μm beam, and 25 ms dwell time. 

Figure 4A clearly shows a comparative lack of Ca in the vasculature of *cax1-1* relative to the wild type Columbia-0, and greater abundance of Ca in the vasculature of 35S-s*CAX*1 (Figure 4A). The images in Figure 4A are shown on individual color scales, with the maximum pixel abundance shown for each line directly beneath it. Performing region of interest analysis, where abundance values are extracted from these regions and used to generate descriptive statistics (mean ± standard deviation), shows these abundance differences quantitatively (Figure 4B). Vascular localization in 35S-s*CAX*1 cotyledons indicates higher transport of Ca into either the vasculature or the bundle sheath—higher resolution analysis of leaf cross sections is required to distinguish the exact cell layer. 

### 4.3. Determine Distribution and Speciation of Ca within Modified Crops

We hypothesize that the intracellular Ca distribution and speciation will be altered in the tissues of Ca-enhanced foods. We plan to image Ca distribution in edible plant tissue using SXRF, and determine the chemical binding form of Ca using the complementary technique of X-ray absorption spectroscopy, and then develop strategies to boost the Ca content of various fruits and vegetables to increase the bioavailable levels of Ca in the plant matrix. The simultaneous multi-elemental capabilities of SXRF are ideal for measuring the effect of gene modification on other micro- or macronutrient elements. For example, upregulation of the critical Fe transport protein IRT1 [68] can also cause an inadvertent increase in cadmium (Cd), because the transporter has a broad substrate range. 

## 5. The Problem, Current Solutions, and Future Directions in Ca Nutrition

With the financial crisis, the challenges facing the real estate industry are greater than they have been in decades [69]. The same could be said for the field of nutritional genomics. There is strong public resistance to this work and funding remains scarce; however, health problems remain that can be effectively treated by dietary therapies [70]. This is, however, a great opportunity for community outreach; the direct involvement of interested or concerned communities in the science that is needed to show the safety and efficacy of biofortified foods. Communication between biologists and the communities we serve is essential for public acceptance of the kind of work described in this article. Some of the first applications of genetic modification (e.g., herbicide tolerant soybean, “Roundup Ready” developed by Monsanto in 1995) have in many ways turned consumers away. A greater understanding of exactly how plants have been modified is urgently needed to help consumers make more informed decisions about the safety of their food. 

The majority of Americans are interested in improving their diets. Evidence suggests that many are changing their diets and attempting to move closer to established dietary recommendations [71], particularly pregnant and nursing women, and those with existing knowledge of dietary sensitivities. However, the direction and magnitude of these changes vary considerably, both among individuals and among food groups. For example, survey data suggest a trend toward lower fat diets. However, the same data show that individuals are not increasing their consumption of fruits and vegetables as recommended, and that the prevalence of obesity is rising [72].

Government assistance can also have some influence on consumer dietary choices [73]. Food assistance programs can affect the amount and the types of foods consumed by low-income populations. However, the long-term changes on the consumer diet, especially after leaving the program, have so far been uncertain [73]. Certainly, greater food expenditure does not necessarily imply a healthier diet. For the more narrowly targeted programs, such as the School Lunch Program, nutrient intake is typically increased (at least while the recipient remains in the program). For example, children in schools with restricted snack availability had significantly higher frequency of fruit and vegetable consumption than children in schools without restricted snack availability [73]. These recent findings suggest that a restrictive snack policy should be part of a multi-faceted approach to improve children’s diet quality [74].

With biotechnology, one hopes to provide substantial benefit to the public at little inconvenience and minimal cost to the consumer [75]. This passive method does not require consumer knowledge, understanding, or commitment to change food consumption behavior.

If biotechnology can be used to increase the dietary Ca levels five-fold in potatoes, lettuce and carrots, we could have a significant effect on total Ca consumption in the United States without needing to alter dietary habits. Currently, Americans receive less than 2% of their Recommended Daily Intake (RDI) of Ca from these vegetables (RDI of 1100 mg Ca per day). The U.S. RDI for Ca is linked to age and stage of life, so among adolescents these vegetables contribute even less to their daily Ca requirements. If Americans continue to eat 60 kg of potatoes a year (approximately 150 medium size potatoes) they would receive a net benefit of 18,000 mg of Ca from these alterations (22,500 in the biofortified potatoes *vs.* 4500 in the varieties found today). For lettuce, if Americans continue to eat 35 lbs of lettuce per year (approximately 200 servings of 1 shredded cup) the net gain in Ca would be 8000 mg from this food (10,000 mg in the biofortified *vs.* 2000 in the standard variety). For carrots, if Americans consume 11 pounds of carrots per year (35 servings of chopped carrots) that would increase Ca consumption by 4100 mg (5250 mg from the biofortified carrots compared with the 1050 mg from standard carrots). These back-of-the-envelope calculations and predictions assume optimal preparation of the vegetables to ensure Ca bioavailability. While bearing in mind these caveats, these Ca-biofortified vegetables could contribute approximately 5% of the RDI of Ca for a wide array of Americans.

## 6. Conclusions

Calcium is an element critical to many body functions. Chronically low Ca intake decreases bone mass and increases the risk of osteoporosis. Currently, the dietary quantities of vegetables required to replace even the amount of Ca in a single glass of milk are difficult to consume on a daily basis. Recently, modified plant-based foods have been developed that remove antinutrients or boost total Ca content. These modified foods have been used in feeding studies to demonstrate improved Ca nutrition. However, the magnitude of the enhanced content exceeds the degree of improved bioavailability. These alterations in the fractional absorption may be caused by changes in localization and speciation of the Ca within the plant matrix. As we suggest throughout his review, for endeavors such as biofortification, it is necessary to emphasize strategies that improve the bioavailable form of the nutrient rather than simply increasing their bulk content. Our long-term goal is to combine genetic engineering and imaging approaches to identify the relationships between nutrient distribution and subsequent changes in the chemical forms of nutrients in the plant cell. This should provide a means of improving bioavailable levels of nutrients in plant-based foods while maximizing crop yield.
